# Crossing boundaries: toward a general model of neuroaesthetics

**DOI:** 10.3389/fnhum.2015.00443

**Published:** 2015-08-07

**Authors:** Manuela M. Marin

**Affiliations:** Department of Basic Psychological Research and Research Methods, University of ViennaVienna, Austria

**Keywords:** neuroaesthetics, crossmodal, multimodal, interdisciplinarity, humanities

Since its dawn in the early 2000s, neuroaesthetics has been flowering as an independent research field (Nadal and Skov, 2013). Its emergence has mostly been driven by researchers who specialize in the study of visual perception and cognition, and who show interest in visual arts. This strong link between neuroaesthetics and vision science is reflected not only by the fact that the term “neuroaesthetics” was coined by the renowned vision researcher Semir Zeki (Zeki and Nash, 1999), but also by recent reviews on neuroaesthetics focusing primarily on aesthetic experiences induced by paintings, abstract patterns, landscapes, faces, architecture, fashion and design objects (Cinzia and Vittorio, 2009; Cela-Conde et al., 2011; Chatterjee, 2011, 2014; Nadal, 2013; Chatterjee and Vartanian, 2014). Current models of aesthetic experiences and their brain correlates revolve around the visual modality and include moderators of aesthetic experiences (social, cultural and situational context, personality, expertise etc.) (Ramachandran and Hirstein, 1999; Chatterjee, 2004; Tinio, 2013; Leder and Nadal, 2014; Redies, 2015). These models are not exclusively concerned with the study of beauty or preference, although these concepts are of historical importance, but include a wide range of aesthetic emotions, judgements, and behaviors.

What may be the underlying reasons for this prevalence of studying the neural underpinnings of visual aesthetic experiences? As in any developing research field, it takes a critical number of researchers who are motivated to organize and attend conferences on a given topic (the first conference on Neuroaesthetics took place in 2009, see Nadal and Pearce, 2011), to write books (Zeki and Nash, 1999; Shimamura and Palmer, 2012; Chatterjee, 2013) and to edit special issues in research journals (Nadal and Skov, 2013). Since the vision science community is relatively large in comparison to other research communities in the field of experimental psychology, it is not unanticipated that several prominent figures were able to attract the attention of younger researchers to help launch this fascinating research field. Another reason for the leading status of visual neuroaesthetics may be that, before the advent of neuroscience, interest in empirical aesthetics was stronger in the vision science community than in other research communities. Therefore, the move toward applying neuroimaging methods to questions related to aesthetic experiences seemed the next logical step. Furthermore, the investigation of aesthetic responses to static images of different kinds may be easier than the study of dynamic images, such as film, video art and the performing arts, and the study of dynamic artforms, such as music and poetry.

Lately, the prolific efforts of the visual neuroaesthetics community have attracted the attention of researchers working outside vision science. For example, the number of publications on music, aesthetics and the brain has clearly increased in the last few years (Brattico and Pearce, 2013; Zatorre and Salimpoor, 2013; Koelsch, 2014b), and a neurocognitive model of the aesthetic experience of music was recently published (Brattico et al., 2013). Similarly, research on the neuroaesthetics of literature reception (Bohrn et al., 2013; Jacobs, 2015) and dance (Calvo-Merino et al., 2008; Cross and Ticini, 2012; Christensen and Calvo-Merino, 2013) has opened new pathways to a deeper understanding of aesthetic experiences. With this in mind, it is very refreshing to see that, for instance, comparative brain imaging studies (Ishizu and Zeki, 2011), metanalyses incorporating different perceptual senses (Brown et al., 2011) and reviews considering different artforms (Nadal, 2013) have been published. Furthermore, an interdisciplinary neuroscience of aesthetic experiences across the three Sister Arts (paintings, poetry, and music) has recently been proposed (Starr, 2013). Undoubtedly, these pioneering attempts may indicate that neuroaesthetics will move away from a field primarily concerned with visual artforms to a field encompassing different artistic modalities in the future. But can this expansion to other artforms be the ultimate goal of the field?

Although the study of aesthetic experiences across different artforms appears a promising avenue because it may lead to broader, overarching theories of aesthetic experiences, such an endeavor may still remain restrictive. The reasons for this are manifold. A neuroaesthetics of the arts is not in line with the initial, broad definition of aesthetics (*scientia cognitionis sensitivae*) by Baumgarten (2007, 1750/1758), but rather based on the notion that aesthetics concerns the study of the arts and beauty. Moreover, Kant already considered human-made art as well as nature in his reflections on aesthetics in his *Critique of Judgment* (1790), thus suggesting a comprehensive approach to aesthetics. In the last third of the twentieth century, philosophy witnessed a revival of this view by the emergence of environmental aesthetics (Carlson, 2000; Budd, 2002). Purely art-based research in empirical aesthetics is also not in line with the influential empirical approach proposed by Berlyne (1960, 1971) in his “new experimental aesthetics,” which conceived aesthetics within the realm of motivation, curiosity, and exploratory behavior in humans and animals. Therefore, a narrow, arts-based approach to neuroaesthetics does not do justice to the frequent induction of aesthetic experiences by objects other than artworks (i.e., natural landscapes, food, faces, bodies, sounds etc.) (Jacobsen, 2006; Brown and Dissanayake, 2009; Tinio, 2013; Zaidel, 2015), but probably also hinders any substantial theoretical developments in the context of evolutionary aesthetics (Grammer et al., 2003). Hence, the neuroaesthetics community will need to follow a comprehensive, broad approach by incorporating the widest possible range of objects unless neuroartsology (Brown and Dissanayake, 2009), i.e., a neuroaesthetics on human art, is in the center of interest. However, this does not entail that neuroaesthetics should become a discipline in which the motto “anything goes” is prevailing. On the contrary, objects of interest should be primarily those for which it has already been shown that they have the capacity to induce aesthetic experiences in humans.

Given that empirical aesthetics in general and neuroaesthetics in particular are lacking a broad theory of aesthetic experiences, which may partly be a byproduct of the general specialization in psychological research after World War II, it is a valid question to ask whether such a general theory of aesthetic experiences and underlying brain correlates should be the goal of the field. In my opinion, the striving for broad, overarching theories, even if they develop out of influential narrow theories such as those on aesthetic responses to human visual artworks, may yield crucial insights into the nature of aesthetic experiences that may otherwise have been missed. Even if extensive efforts devoted to advance a general theory of aesthetic experiences eventually fail, insights into the specificities of aesthetic experiences would not become apparent if aesthetic experiences were not systematically compared among different types of external objects (based on perception) and internal objects (based on thoughts and mental imagery). In other words, the question of whether specific aesthetic theories (e.g., those restricted to art) are justified can only be answered by trying to falsify the findings obtained for one type of object. As an illustration, inventive studies such as those comparing the processing of moral and facial beauty (Wang et al., 2015) or beauty induced by paintings and music (Ishizu and Zeki, 2011) should be highlighted. In a similar vein, neuroimaging studies investigating the beauty of mathematical formulas (Zeki et al., 2014) are equally encouraging.

How could such an ambitious goal of a general theory of neuroaesthetics be accomplished in the long run? One possibility is to further develop the study of other basic senses than vision, that is, to expand the *sensory-based* approach to empirical aesthetics. In general, it is not known yet whether effects reported for (different types of) visual objects generalize to other sensory domains. Therefore, a systematic comparative approach could offer new insights into meta-sensory findings and those that are unique for a sense. Such an approach does not always entail the use of different types of sensory stimuli within one research design, but also a replication of effects found for the visual domain with other sensory domains. For example, does the often reported mere-exposure effect (Zajonc, 1968) hold true for all types of sensory objects categories (Bornstein, 1989)? And more specifically, are the neural correlates of this effect, like those recently shown for faces (Kongthong et al., 2014), same or different across sensory domains and objects? Notably, the study of haptic perception in relation to aesthetic experiences has already yielded some promising results by following this research strand (Hintz and Nelson, 1971; Ballesteros and Reales, 2004; Jansson-Boyd and Marlow, 2007; Juricevic, 2009; Jakesch et al., 2011; Jakesch and Carbon, 2012; Etzi et al., 2014), which have recently not only led to initial speculations of underlying brain correlates (Gallace and Spence, 2011) but also to the development of a model of haptic aesthetic processing (Carbon and Jakesch, 2013).

As implied above, moving toward a general theory of neuroaesthetics will also require the systematic comparison of different object classes *within* one sensory domain, which is already ongoing to some degree (Vessel et al., 2014). However, there exists a vast array of possibilities of how to categorize objects within one sensory modality. In the visual domain, categories may range from human vs. non-human, human artwork vs. non-artwork, static vs. dynamic, utilitarian vs. decorative, to abstract vs. representational, to name only a few possible categories. The brain correlates of different object classes in relation to aesthetic experiences have already been partly compared in the visual domain (Cela-Conde et al., 2004; Kawabata and Zeki, 2004; Cattaneo et al., 2014), but this endeavor could be more rigorously followed and expanded to other sensory domains.

Another slowly growing research path is concerned with the direct comparison of perceptual, cognitive and affective processes *across* sensory domains within one research design. Such an approach is particularly valuable because it is mostly driven by concrete cross-domain research questions, often applies implicit or indirect tests, allows for a stricter control of participant characteristics, and importantly, does not ignore the fact that we live in a multimodal world with multimodal artforms. In fact, there is a wide variety of recent behavioral and neurophysiological evidence against the notion of strict modularity of sensory modalities in humans (De Gelder and Bertelson, 2003; Shams and Kim, 2010; Gerdes et al., 2014) and animals (Van Wassenhove et al., 2012), which challenges the long-standing view of studying perception (and action) primarily from a unimodal perspective. In the field of empirical aesthetics, the small number of studies that follows this attitude does not take human artworks as a starting point for their investigations. Yet psychological concepts that have proven to be relevant for theories of aesthetic experiences, such as complexity (Boon et al., 2011; Marin and Leder, 2013), are currently examined across domains. Furthermore, aesthetic emotions in different artforms have been extensively studied (Silvia, 2005; Juslin, 2013; Koelsch, 2014a), but studies with crossmodal or multimodal designs are still outnumbered except for those on emotional pictures and sounds (e.g., Baumgartner et al., 2006a,b; Spreckelmeyer et al., 2006; Logeswaran and Bhattacharya, 2009; Marin and Bhattacharya, 2011; Petrini et al., 2011; Marin et al., 2012; Gerdes et al., 2014). A related issue concerns one of the core concepts of the field, namely beauty, and how it may differ in artforms (Augustin et al., 2012), types of objects (Marković, 2014), and neural processes (Ishizu and Zeki, 2011). Hopefully these valuable comparative studies will inspire researchers to continue working along these lines.

It must be remembered that a comparative approach to neuroaesthetics requires knowledge that may go beyond the expertise of an individual researcher, who usually works on a specialized research topic within a subfield of experimental psychology or cognitive neuroscience (e.g., emotion, visual, auditory and haptic perception, multisensory processing, psycho- and neurolinguistics, numerical cognition etc.). Clearly, collaborations of researchers with different backgrounds may yield better results. I also see much potential in a tighter collaboration between the humanities (musicology, art history, literary studies, dance studies, media and communication studies, semiotics, linguistics, philosophy, and mathematics) and the sciences (psychology, biology, and neuroscience) to gain a better understanding of aesthetic experiences. People trained in the humanities have the ability to detect aesthetically relevant phenomena by analytical thinking and abstraction. This usually goes hand in hand with the development of an appropriate terminology, classification system, and theoretical framework. For instance, the comparative study of human artforms has led to a research field called intermediality studies (Rajewsky, 2005), in which specific characteristics of media as well as their complex relations are the focus of interest. This field has already provided valuable theoretical insights into phenomena that contribute to people's aesthetic experiences across media such as music, literature and the visual arts (Wolf and Bernhart, 2006, 2007, 2013). However, these theories have not been subjected to empirical research methods yet. The humanities' awareness of the historical, social and cultural context in which artworks are embedded constitutes another asset that the sciences unfortunately often lack (Bullot and Reber, 2013; Redies, 2015). Importantly, intermediality studies could potentially be extended beyond the realm of arts and offer a refined alternative to the investigation of aesthetic experiences by going beyond the study of beauty and preference (Chatterjee and Vartanian, 2014; Consoli, 2015).

All things considered, I believe that advances in neuroaesthetics can be made by attracting more scientists and humanists from outside the visual aesthetics community, by comparing diverse aesthetic experiences across sensory modalities, and by giving up the notion that aesthetics concerns exclusively the study of beauty and the arts. Based on the knowledge generated by this comprehensive and comparative approach to neuroaesthetics, the study of top-down influences and moderators of aesthetic experiences as well as of aesthetic experiences induced by internal objects may become easier to accomplish from a neuroscientific perspective in the future.

## Conflict of interest statement

The author declares that the research was conducted in the absence of any commercial or financial relationships that could be construed as a potential conflict of interest.
